# Primary Jejunal Enterolith Causing Small Bowel Obstruction Without Any Underlying Bowel Abnormality

**DOI:** 10.7759/cureus.28743

**Published:** 2022-09-03

**Authors:** Evgenia Karveli, Ciaran Barlow, Charlotte Grant, Soraya Conroy, Michail Papamichail

**Affiliations:** 1 General Surgery, Evangelismos General Hospital, Athens, GRC; 2 General Surgery, The Royal United Hospital Bath, Bath, GBR

**Keywords:** jejunal diverticula, idiopathic, obstruction, small bowel, enterolith

## Abstract

Enterolith formation is a rare condition precipitated by decreased bowel motility. It may cause obstruction or other complications and the diagnosis usually is confirmed after surgery and analysis of the stones or fragments. It is often seen in association with intestinal abnormalities such as diverticula and inflammation or in biliary tract fistulas where stones migrate to the duodenum and small bowel. We report an unusual case of a primary true enterolith formation in a patient without any underlying bowel condition or any previous surgery.

## Introduction

Primary enterolith formation in the duodenum and small bowel is rare and is usually associated with conditions such as diverticular disease, strictures, previous surgery, and chronic inflammation, that cause decreased motility and accumulation of certain matters leading eventually to stone or fragments formation [1,2]. In addition gallbladder or bile duct and renal tract fistulas are causes of secondary enteroliths when a stone(s) that has formed in a different organ migrates to the duodenum or small bowel. Finally, oral intake of indigestible materials (bezoars) or insoluble liquids can interact with intestinal contents and create solid particles (false enteroliths) [3].

The reported prevalence varies from 0.3% to 10% and is related to etiology, and underlying risk factors [3]. Patients with enteroliths are often asymptomatic, but when size is > 2-3 cm, they are at risk of developing obstruction or other complications. Diagnosis may be suspected from imaging findings in relation with the patient’s symptomatology, but confirmation is usually followed after surgery when the enterolith is extracted and the associated intestinal pathology can be assessed [4]. We report an unusual case of a patient with small bowel obstruction due to enterolith impaction in the jejunum without any previous surgery or other gastrointestinal abnormality.

## Case presentation

**Figure 1:**
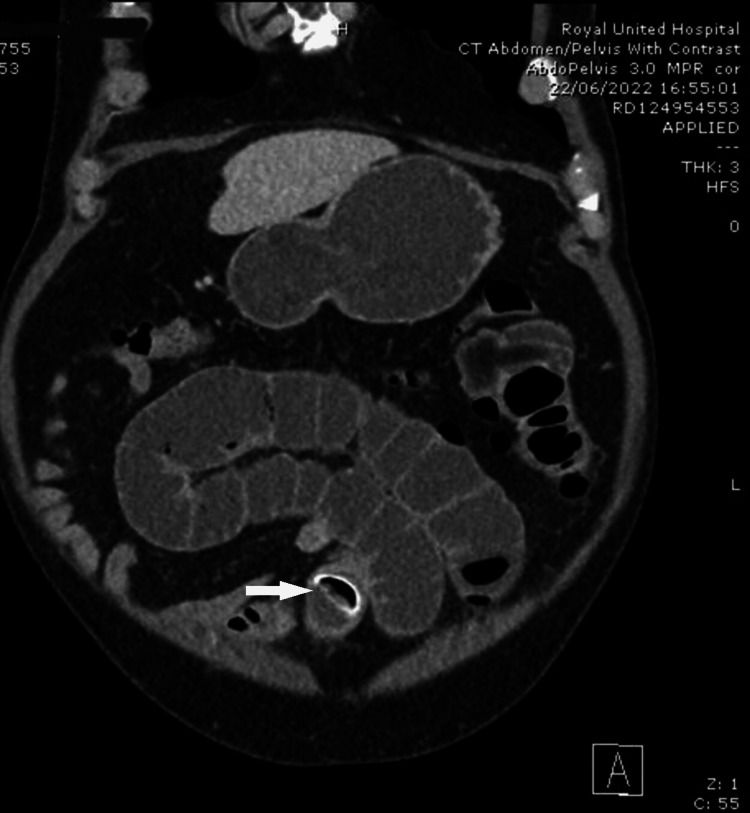
Computed tomography image White arrow showing intraluminal foreign body causing small bowel obstruction

A 69-year-old male with no significant past medical history, presented with a 24-hour history of sharp, lower abdominal pain, nausea and vomiting, and failure to pass stool or flatus in ~48 hours. His inflammatory markers were mildly elevated (white cells 13,4 × 10^9^/L and C-reactive protein 211 mg/L). Imaging revealed a small bowel obstruction with evidence of a 3x4 cm foreign body in the distal jejunum causing upstream dilatation (Figure 1).

**Figure 2:**
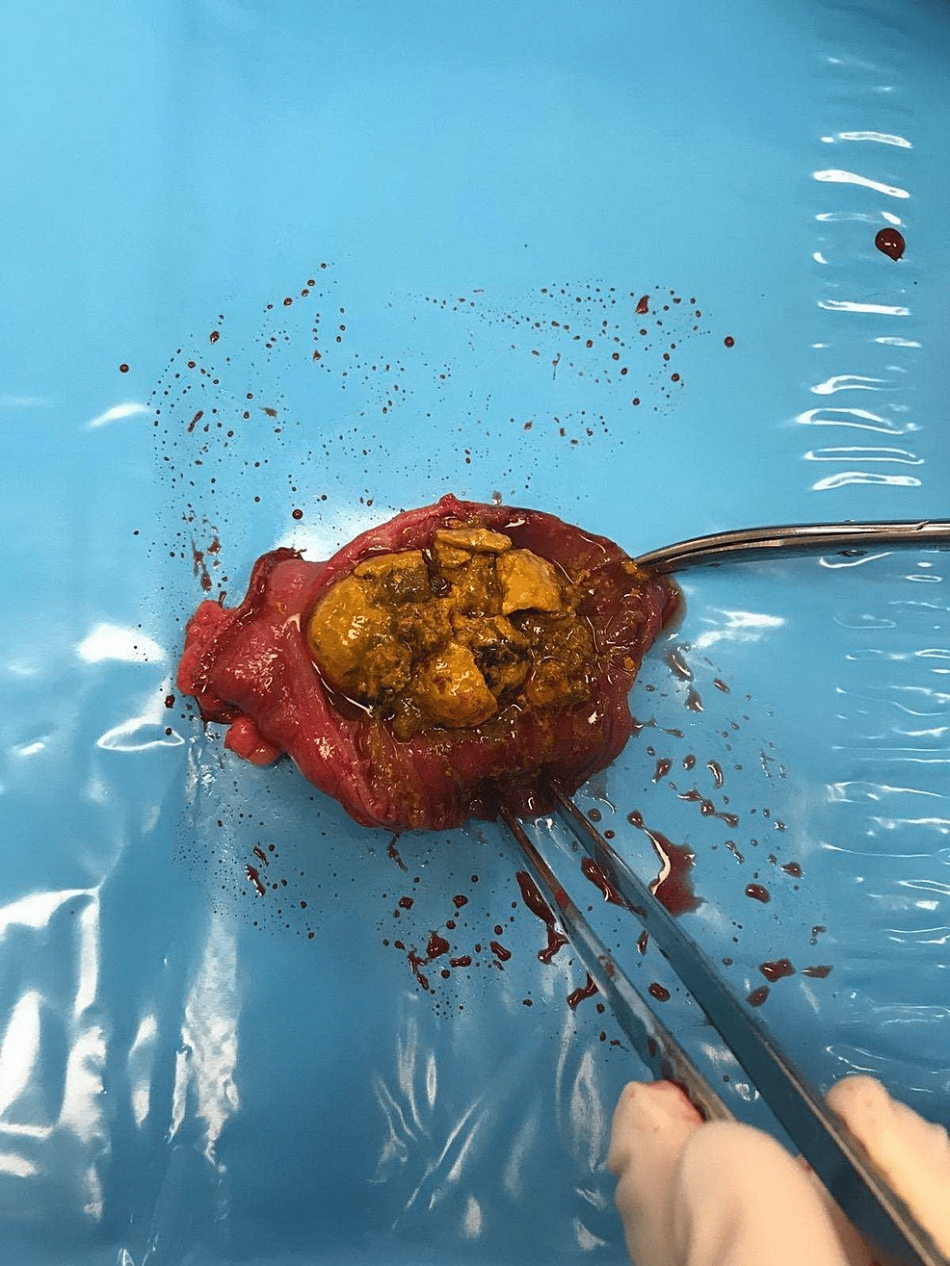
Resected small bowel containing enterolith

There was no abnormality in other organs including the gallbladder, duodenum and large bowel, nor was there any underlying disease in the rest of the jejunum and ileum. The patient had no previous abdominal surgery. Primary differential diagnosis included bezoar but the patient denied having swallowed anything unusual. Initially he was treated conservatively and subsequent gastrografin follow through showed evidence of passing to the colon. However, the patient continued having intermittent vomiting and could not pass flatus after four days from admission. A repeated computed tomography showed persistent jejunal dilatation and the foreign body had migrated only a short distance distally. A decision was made to perform exploratory laparotomy for diagnosis and definitive treatment. With a midline incision an impacted foreign body, of about 4 cm in size, was discovered in the distal jejunum and a segmental (10 cm) small bowel resection with side-to-side jejunojejunostomy was performed. There was no associated small bowel pathology on gross examination and the gallbladder was normal on visualization and palpation. The resected bowel was opened and a cluster of fragmented stones was extracted. They appeared hard in consistency with tendency to crumble into smaller pieces after manual handling (Figure 2).

Given the fact that there was no evidence of biliary pathology nor any findings of indigestible or insoluble material, there was very strong suspicion for those stones to be an enterolith. Histology did not reveal any pathological intestinal tissue but stone analysis was not possible. The patient had an uneventful recovery and was discharged five days later. There was no evidence of underlying abdominal pathology that could potentially explain the mechanism of enterolith formation on this patient.

## Discussion

Intestinal hypo motility or prolonged stasis is the most common predisposing factor for primary enterolith formation. Causes include diverticular disease (e.g. Meckel’s diverticulum), areas of anastomoses, blind pouches, afferent loops, adhesions, and inflammatory strictures (e.g. Crohn’s disease, radiation, tuberculosis) [3]. Intraluminal stasis promotes bacterial overgrowth, resulting in bile salt deconjugation precipitating concretion, and eventually the formation of solid particles. The usual contents include fecal matter, calcium phosphates, magnesium, bacteria, debris, and unconjugated bile acids [4,5]. Choleic acid enteroliths require lower pH and are most commonly seen in the duodenum and the proximal small bowel as opposed to calcium salt stones that form in an alkaline environment usually in the distal ileum [6,7]. False enteroliths such as bezoars form during their passage through the intestinal lumen inspissating with enteral contents [5]. Biliary tract fistulas with migrated gallstones remain the most common form of secondary enterolithiasis and usually becomes apparent when complicated with ileus. In general, etiology typically follows gender and age predisposition of the underlying condition [3].

Enteroliths may vary in size, number, and configuration ranging from a few millimeters to 10 cm, as a single stone or cluster of fragments [3]. Symptoms likely occur with larger enteroliths in a setting of obstruction or another complication or may be related to the underlying intestinal abnormality precipitating the enterolith formation (e.g. chronic inflammation) [6]. Imaging such as plain films, contrast studies, and computed tomography may be helpful in diagnosis and also reveal possible associated intestinal pathology. Confirmation usually follows surgical intervention in symptomatic and complicated patients [4]. In our case computed tomography suggested small bowel obstruction due to an intraluminal foreign body whose nature was uncertain given the absence of any other gastrointestinal or biliary pathology. The presence of stones inside the jejunum raised strong suspicion that this is an enterolith. 

Treatment is usually surgical and is indicated in patients presenting with obstruction, perforation, or other complications. The type of surgery is directed by the associated pathology. Surgical options include digital fragmentation of the stones and manual propelling into the large bowel, enterotomy, and stone extraction or small bowel resection. Resection is commonly performed in the presence of diverticula, severe inflammation or strictures, necrosis, and perforation [8,9]. The use of endoscopic retrieval or lithotripsy has also been described for duodenal enteroliths [10].

Similar to our case, the formation of primary enterolith or stone material inside the small bowel without any precipitating factor has been reported in the past, however, the mechanism remains unclear [1,6,7]. In one case the enterolith contained uric acid but again without any association with any other anatomical, functional or biochemical abnormality (e.g hyperuricemia) [7]. Shrestha and Shrestha have reported on one patient who had an episode of “idiopathic” obstructive enterolith formation followed by surgery and in the future had two more episodes of obstruction due to recurrent entrolithiasis possibly due to adhesions [4]. In such cases enterolith analysis when possible may be useful in indicating a possible mechanism or particular area of formation. In our case, there was no evidence of any indigestive or insoluble material and further analysis could have provided more details about the contents of the enterolith and confirmed the diagnosis. However, macroscopically there was such strong evidence that those fragmented stones were enteroliths. It is unclear if those patients may have an idiopathic subclinical bowel dysmotility which predisposes them to this condition. Our patient has not been tested for any bowel dysmotility in the past and has reported normal bowel habits which makes a very rare case of stone formation in the jejunum. 

## Conclusions

In conclusion, enterolithiasis is a rare cause of intestinal obstruction and sometimes can present without any predisposing factor. Surgery can provide definitive diagnosis and treatment. Surgeons should be careful, while dealing with cases of acute intestinal obstruction and consider less common causes in their differential diagnosis including enteroliths and dysfunctional bowel motility. Obtaining detailed history is very important to identify potential causes of enterolith formation and should always include the possibility of foreign body ingestion. Analysis of the enterolith and associated bowel pathology may be useful to identify the potential mechanism of formation. 

## References

[1] Abtar HK, Mneimneh M, Hammoud MM, Zaaroura A, Papas YS (2016). Primarily proximal jejunal stone causing enterolith ileus in a patient without evidence of cholecystoenteric fistula or jejunal diverticulosis. Case Rep Surg.

[2] Seretis C, Archer L, Elhassan MA, Gurung D, Palit A, Zayyan K (2021). Small bowel obstruction secondary to primary enterolith: a rare and delayed complication of radiation enteritis. Clin Case Rep.

[3] Gurvits GE, Lan G (2014). Enterolithiasis. World J Gastroenterol.

[4] Shrestha AL, Shrestha P (2017). Recurrent enterolithiasis small bowel obstruction: a case seldom described. Case Rep Gastrointest Med.

[5] Lee MC, Bui JT, Knuttinen MG, Gaba RC, Scott Helton W, Owens CA (2009). Enterolith causing afferent loop obstruction: a case report and literature review. Cardiovasc Intervent Radiol.

[6] Jadib A, Tabakh H, Chahidi El Ouazzani L (2022). Primary true enterolithiasis: a rare cause of acute small bowel obstruction. Radiol Case Rep.

[7] Quazi MR, Mukhopadhyay M, Mallick NR, Khan D, Biswas N, Mondal MR (2011). Enterolith containing uric acid: an unusual cause of intestinal obstruction. Indian J Surg.

[8] Wauters L, Peeters K, Van Hootegem A, Goetstouwers P, Delvaux P, Callens J (2018). Meckel's enterolith : a rare cause of mechanical small bowel subobstruction. Acta Gastroenterol Belg.

[9] Ferrari-Light D, Shuchleib A, Ricci-Gorbea J (2018). Bile salt enterolith: an unusual etiology mimicking gallstone ileus. Case Rep Surg.

[10] Patel C, Balasubramaniam R, Bullen T (2020). Jejunal enterolith: a rare case of small bowel obstruction. Cureus.

